# Aberrant DNA methylation reprogramming in bovine SCNT preimplantation embryos

**DOI:** 10.1038/srep30345

**Published:** 2016-07-26

**Authors:** Sheng Zhang, Xin Chen, Fang Wang, Xinglan An, Bo Tang, Xueming Zhang, Liguang Sun, Ziyi Li

**Affiliations:** 1 State & Local Joint Engineering Laboratory for Animal Models of Human Diseases, Academy of Translational Medicine, First Hospital, Jilin University, Changchun, Jilin 130061, China; 2 College of Animal Science and Veterinary Medicine, Jilin University, Changchun, Jilin 130062, China; 3 Air Force General Hospital of PLA, Beijing 100037, China

## Abstract

DNA methylation reprogramming plays important roles in mammalian embryogenesis. Mammalian somatic cell nuclear transfer (SCNT) embryos with reprogramming defects fail to develop. Thus, we compared DNA methylation reprogramming in preimplantation embryos from bovine SCNT and *in vitro* fertilization (IVF) and analyzed the influence of vitamin C (VC) on the reprogramming of DNA methylation. The results showed that global DNA methylation followed a typical pattern of demethylation and remethylation in IVF preimplantation embryos; however, the global genome remained hypermethylated in SCNT preimplantation embryos. Compared with the IVF group, locus DNA methylation reprogramming showed three patterns in the SCNT group. First, some pluripotency genes (*POU5F1* and *NANOG*) and repeated elements (satellite I and α-satellite) showed insufficient demethylation and hypermethylation in the SCNT group. Second, a differentially methylated region (DMR) of an imprint control region (ICR) in *H19* exhibited excessive demethylation and hypomethylation. Third, some pluripotency genes (*CDX2* and *SOX2*) were hypomethylated in both the IVF and SCNT groups. Additionally, VC improved the DNA methylation reprogramming of satellite I, α-satellite and *H19* but not that of *POU5F1* and *NANOG* in SCNT preimplantation embryos. These results indicate that DNA methylation reprogramming was aberrant and that VC influenced DNA methylation reprogramming in SCNT embryos in a locus-specific manner.

DNA methylation is an important epigenetic modification that occurs predominantly at CpG dinucleotides. It is involved in a number of key genomic functions, such as gene imprinting, X chromosome inactivation, genome stability, retrotransposon silencing and gene inactivation in cancer^1^,^2^,^3^. DNA methylation is catalyzed by members of the DNA methyltransferase (DNMT) family. The DNMT family mainly consists of three members: DNMT1, DNMT3a and DNMT3b. DNMT3a and DNMT3b are called *de novo* DNA methyltransferases and are responsible for the initial establishment of new DNA methylation patterns^4^,^5^. DNMT1 plays an important role in the faithful maintenance of DNA methylation patterns during DNA replication^6^,^7^.

The removal of DNA methylation is termed DNA demethylation. DNA demethylation can occur by two different mechanisms. The first, termed “active” demethylation, occurs rapidly and independent of cell division and is catalyzed by unknown enzymes that cleave the methyl group^8^. The other mechanism, called “passive” demethylation, occurs when DNA methylation is passively diluted by DNA replication following cell division due to the absence of the maintenance methyltransferase DNMT1^9^. Ten-eleven translocation (TET) family, which includes TET1, TET2 and TET3, is generally believed to play important roles in the progression of active demethylation. Recent studies have found that DNA demethylation occurs via a combination of active and passive demethylation^10^,^11^,^12^.

DNA methylation patterns are obviously reprogrammed in mammalian preimplantation embryos^13^. Genetic expression analysis indicates that *Tet3*, but not *Tet1* and *Tet2*, is expressed at high levels in oocytes and zygotes but rapidly decreases beginning at the 2-cell stage^14^,^15^. TET3 can oxidize 5-methylcytosine (5-mC) to 5-hydroxymethylcytosine (5-hmC), 5-formylcytosine (5-fC) and 5-carboxylcytosine (5-caC) during the active demethylation of the paternal genome at the zygote stage^10^,^11^,^16^,^17^,^18^. The oxidized derivatives of 5-mC are further passively diluted by DNA replication during early cell division^12^. Through either the conditional deletion of *Tet3* from the female germ cells or the siRNA-mediated down-regulation of zygotic *Tet3*, studies have found that TET3 is responsible for the conversion of 5-mC to 5-hmC in the paternal genome^15^,^16^. The deletion of *Tet3* causes an increased frequency of developmental failure in embryos^16^. The above results suggest the importance of proper TET3-catalyzed DNA methylation reprogramming in normal mammalian early embryonic development.

Somatic cell nuclear transfer (SCNT) is a technique by which differentiated cells can be converted to the totipotency state through a mechanism that depends on the reprogramming of epigenetic modifications. Despite success in cloning various animal species, the use of somatic cells as the source of donor nuclei has raised many practical and relevant concerns, such as increased abortion rates, high birth weights and perinatal death^19^,^20^,^21^. The anomalies associated with SCNT embryos may be caused by the incomplete reprogramming of epigenetic modifications in the somatic cell nucleus of an enucleated oocyte that involves the normal transcriptional reactivation of embryonically expressed genes^22^,^23^. The reprogramming of DNA methylation during normal mouse fertilization and SCNT embryonic development is partially understood^14^,^15^,^16^; however, the changes that occur during embryonic development vary among species^23^. Thus, it is important to investigate the reprogramming of DNA methylation in other species to expand our understanding of the mechanism responsible for the abnormal development of SCNT embryos.

Vitamin C (VC), a general antioxidant, is responsible for maintaining the catalytic activity of a group of iron- and 2-oxoglutarate-dependent dioxygenases^24^. Previous studies have shown that VC can enhance somatic cell reprogramming during the generation of induced pluripotent stem cells (iPSCs)^25^. VC is also beneficial for the enhancement of the *in vitro* and *in vivo* development of porcine SCNT embryos^26^. However, the influence of VC on the development of and DNA methylation reprogramming in bovine SCNT embryos is still unknown.

It is reported that DNA methylation between *in vivo* embryos and IVF embryos showed no significant difference^27^, so IVF embryos were used as a control to analyze DNA methylation of SCNT embryos in this study. A previous study showed that there was no difference in DNA methylation in the intragenic DMR within the bovine *IGF2* gene between bovine IVF and *in vivo* blastocysts^28^. To reveal the mechanisms of the abnormal development of SCNT embryos, this study investigated the reprogramming of DNA methylation during bovine IVF and SCNT preimplantation embryonic development and examined the influence of VC on the development of and DNA methylation reprogramming in bovine SCNT preimplantation embryos.

## Results

### IF staining for 5-mC and 5-hmC in IVF and SCNT preimplantation embryos

**Figure 1:**
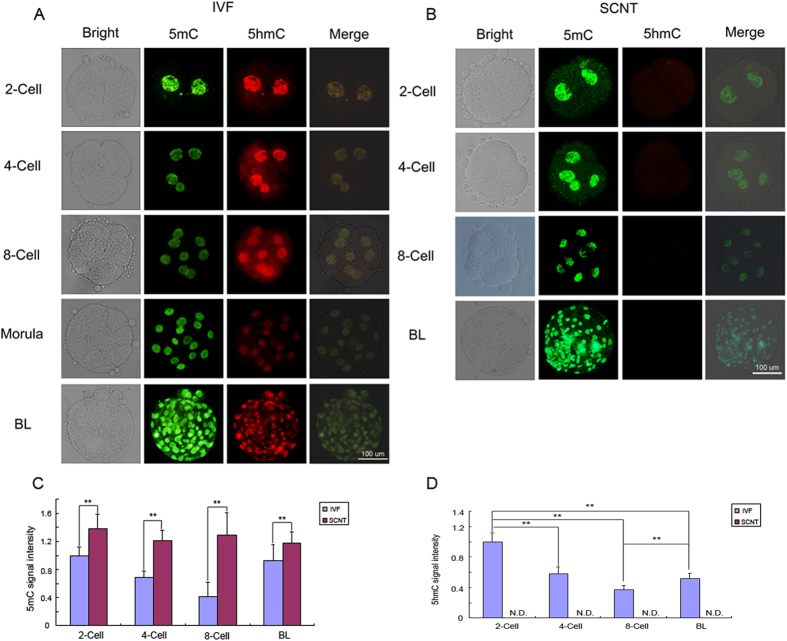
Immunofluorescent (IF) staining of 5-mC and 5-hmC in bovine oocytes and IVF and SCNT preimplantation embryos. (**A**) The distribution of 5-mC (red) and 5-hmC (green) in bovine IVF preimplantation embryos. (**B**) The distribution of 5-mC and 5-hmC in bovine SCNT preimplantation embryos. (**C**,**D**) The 5-mC and 5-hmC signal intensities were measured in at least 5 embryos at each developmental stage. The signal intensity in IVF 2-cell embryos was used as a calibrator sample (set to 1). The results represent the mean ± standard deviation of five independent experiments. **P < 0.01. BL: IVF blastocysts.

The reprogramming of global DNA methylation during bovine IVF and SCNT preimplantation embryonic development was analyzed by immunofluorescent (IF) staining for 5-mC and 5-hmC. IF staining indicated that the 2-cell embryos showed strong IF signals for 5-mC. The signal gradually decreased until 8-cell embryos were formed, and then increased until the blastocyst stage during bovine IVF preimplantation embryonic development. In IVF blastocysts, both inner cell mass (ICM) and trophectoderm cells were methylated. IF staining for 5-hmC showed that 5-hmC was present in all bovine IVF preimplantation embryos. 5-hmC was also observed in both ICM and trophectoderm cells, and 5-mC was observed in bovine IVF blastocysts (Fig. 1A,C,D). However, each bovine SCNT preimplantation embryo showed stronger IF signals for 5-mC than the corresponding IVF preimplantation embryos, and the 5-mC signal did not show obvious changes in the bovine SCNT preimplantation embryos. More interestingly, no 5-hmC signal was observed in any of the developmental stages of the bovine SCNT preimplantation embryos (Fig. 1B,C).

### Methylation of satellite I and α-satellite

**Figure 2:**
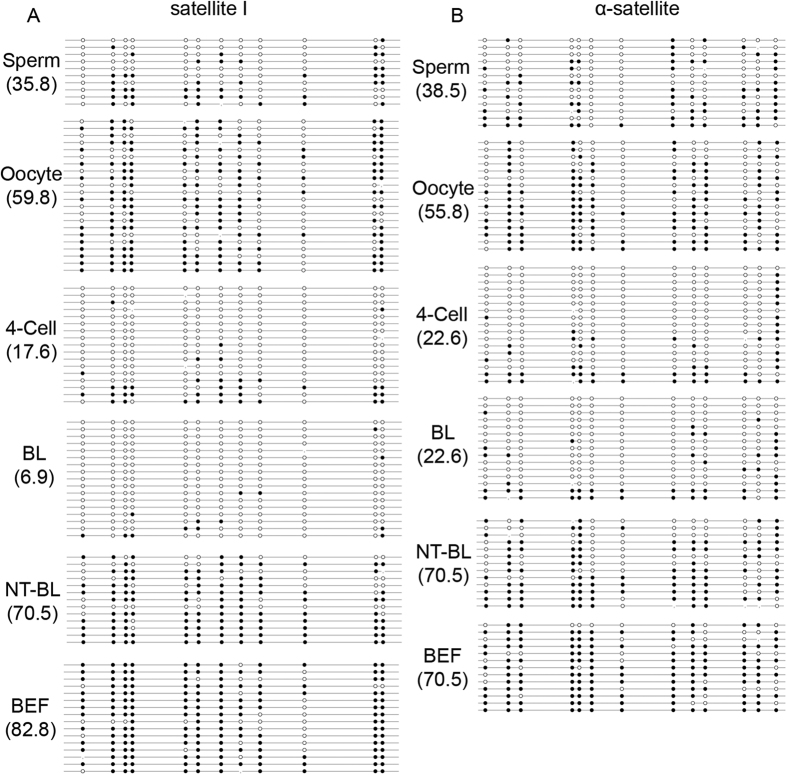
Dynamic DNA methylation profiles of satellite I and α-satellite in bovine gametes, IVF preimplantation embryos, SCNT blastocysts and BEFs. (**A**) Satellite I showed hypermethylation in gametes. After fertilization, the zygotic genome lost DNA methylation in 4-cell embryos, and the DNA methylation levels further decreased in blastocysts. However, BEFs and SCNT blastocysts maintained a high level of satellite I methylation. (**B**) α-satellite showed slight DNA demethylation from gametes to IVF blastocysts and hypermethylation in BEFs and SCNT blastocysts. The filled (black) circles correspond to methylated cytosines, the unfilled (white) circles indicate unmethylated cytosines and the small vertical lines without a circle correspond to missing values. The number denotes the percentage of methylated cytosines observed at all of the CpG sites. BL: IVF blastocysts; NT-BL: SCNT blastocysts; and BEFs: bovine embryonic fibroblasts.

Satellite I and α-satellite were selected as the repeat elements to be tested for DNA methylation reprogramming in bovine IVF and SCNT preimplantation embryos. Bisulfite sequencing results showed that satellite I had a moderate DNA methylation level in sperm (35.8 ± 2.5%) and a high DNA methylation level in MII oocytes (59.8 ± 4.5%). Furthermore, the level of DNA methylation significantly decreased in IVF 4-cell embryos (17.6 ± 3.2%) and then decreased even further in IVF blastocysts (6.9 ± 2.5%). However, satellite I was highly methylated in bovine embryonic fibroblasts (BEFs) (82.8 ± 4.5%) and SCNT blastocysts (70.5 ± 4.7%) (Fig. 2A). The results also revealed that satellite I was insufficiently demethylated and hypermethylated in the SCNT blastocysts (82.3% ± 3.7%) when granulosa cells (88.0 ± 5.6%) were used as the donor cells (Supplementary Fig. 5), indicating that the dynamic DNA methylation changes in the SCNT preimplantation embryos were not donor cell-specific.

**Figure 3:**
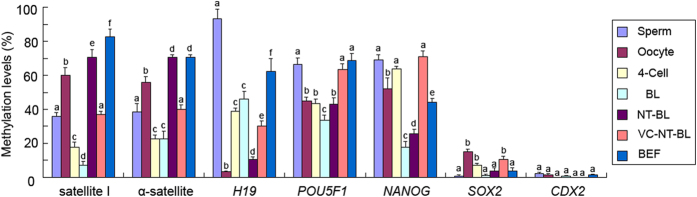
A diagram showing the differences in methylation levels of seven genes in bovine sperm, oocytes, IVF 4-cell embryos, IVF blastocysts, SCNT blastocysts, SCNT blastocysts treated with vitamin C and BEFs. The results represent the mean ± standard deviation of three independent experiments in which at least fifty cells or blastomeres were analyzed. For each gene, different lowercase letters denote a significant difference (*P* < 0.05), and the same letter represents no significant difference (*P* > 0.05). 4-cell: IVF 4-cell; BL: IVF blastocyst; NT-BL: SCNT blastocyst; VC-NT-BL: SCNT blastocyst treated with vitamin C; and BEF: bovine embryonic fibroblasts.

α-Satellite showed a moderate level of DNA methylation in sperm (38.5 ± 4.7%) and a high level of DNA methylation in MII oocytes (55.8 ± 3.4%). The moderate level of DNA methylation slightly decreased in IVF 4-cell embryos (22.6 ± 2.1%) and then persisted until the blastocyst stage of development (22.6 ± 4.5%) following bovine IVF preimplantation. α-Satellite was highly methylated in both BEFs (70.5 ± 1.5%) and SCNT blastocysts (70.5 ± 1.5%) (Figs 2B and 3).

### Methylation of *H19*

**Figure 4:**
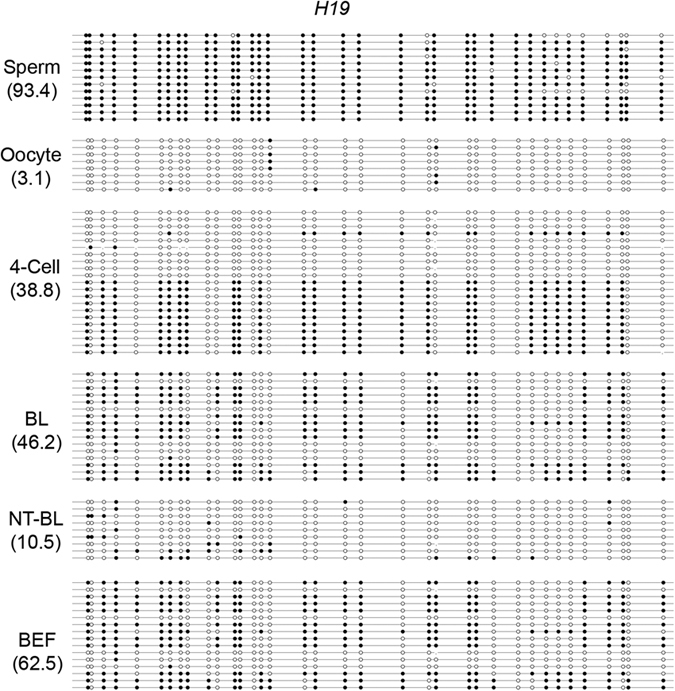
DNA methylation status in the DMR of the bovine *H19* locus. Sperm was hypermethylated, while oocytes were hypomethylated. Moderate methylation was maintained in IVF preimplantation embryos and BEFs. In contrast, SCNT blastocysts were hypomethylated. BL: IVF blastocysts; NT-BL: SCNT blastocysts; and BEFs: bovine embryonic fibroblasts.

We selected one DMR in the *IGF2/H19* locus as a representative imprinted gene to test DNA methylation reprogramming during bovine IVF and SCNT preimplantation embryonic development. The results showed that the DMR was highly methylated in sperm (93.4 ± 5.5%) but hypomethylated in MII oocytes (3.1 ± 0.7%). Moderate DNA methylation was maintained in bovine IVF 4-cell embryos (38.8 ± 1.9%) and blastocysts (46.2 ± 4.5%), but high DNA methylation was observed in BEFs (62.5 ± 7.5%). The DMR DNA methylation level significantly decreased in bovine SCNT blastocysts (10.5 ± 1.8%) compared to BEFs (62.5 ± 7.5%) (Figs 3 and 4).

### Methylation of pluripotency genes

Pluripotency genes, such as *POU5F1*, *NANOG*, *SOX2* and *CDX2*, play important roles in the segregation and maintenance of embryonic and extraembryonic tissues^29^. Therefore, we analyzed the DNA methylation status and mRNA expression levels of these pluripotency gene promoter regions in oocytes, BEFs, bovine IVF preimplantation embryos and bovine SCNT blastocysts. We found that the pluripotency genes could be divided into two groups based on the results.

**Figure 5:**
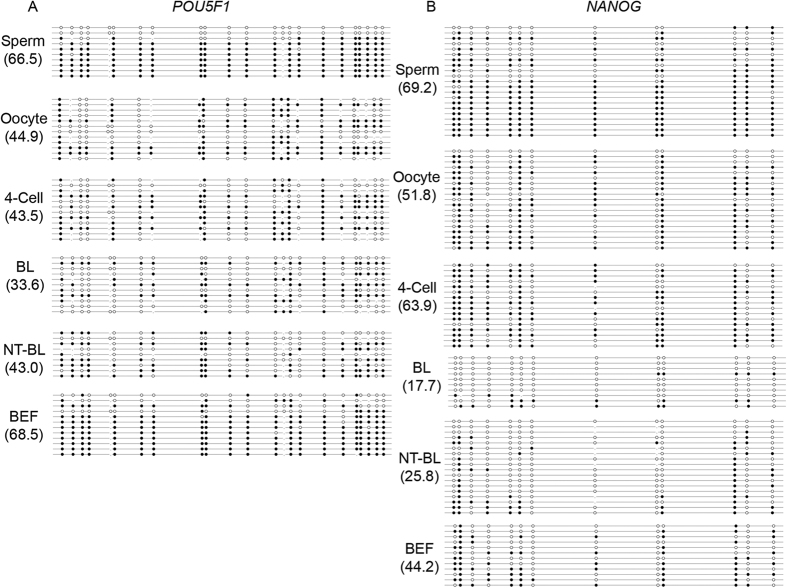
Dynamic DNA methylation profiles of *POU5F1* and *NANOG* in bovine gametes, IVF preimplantation embryos, SCNT blastocysts and BEFs. (**A**) *POU5F1* showed low levels of DNA demethylation from gametes and BEFs to IVF and SCNT blastocysts. (**B**) *NANOG* showed lower DNA methylation levels in IVF and SCNT blastocysts than in gametes and BEFs. BL: IVF blastocysts; NT-BL: SCNT blastocysts; and BEFs: bovine embryonic fibroblasts.

The first group, consisting of *POU5F1* and *NANOG*, followed a DNA demethylation pattern during bovine IVF preimplantation embryonic development. The DNA methylation levels in IVF blastocysts (33.6 ± 2.8% and 17.7 ± 3.5%) were lower than those in sperm (66.5 ± 3.8% and 69.2 ± 2.9%), oocytes (44.9 ± 2.3% and 51.8 ± 6.5%) and 4-cell embryos (43.5 ± 2.5% and 63.9 ± 1.5%). Furthermore, the DNA methylation levels in SCNT blastocysts (43.0 ± 3.9% and 25.8 ± 2.5%) were lower than those in BEFs (68.5 ± 4.5% and 44.2 ± 2.3%) but higher than those in IVF blastocysts (Figs 3 and 5). The mRNA expression levels of *POU5F1* and *NANOG* in IVF and SCNT blastocysts were significantly higher than those in BEFs and other preimplantation embryos (*P* < 0.01), but the mRNA expression levels in SCNT blastocysts were significantly lower than those in IVF blastocysts (*P* < 0.01) (Supplementary Fig. 6A,B).

**Figure 6:**
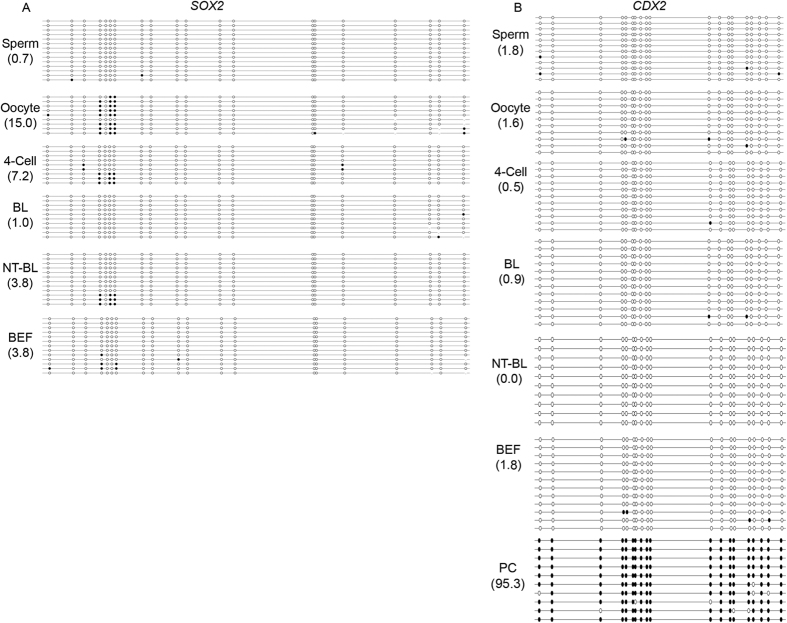
Dynamic DNA methylation profiles of *SOX2* and *CDX2* in bovine gametes, IVF preimplantation embryos, SCNT blastocysts and BEFs. (**A**) *SOX2* and (**B**) *CDX2* were always hypomethylated in bovine gametes, IVF preimplantation embryos, SCNT blastocysts and BEFs. BL: IVF blastocysts; NT-BL: SCNT blastocysts; BEFs: bovine embryonic fibroblasts; and PC: positive control.

The genes in the second group, *SOX2* and *CDX2*, were hypomethylated throughout the development of bovine IVF preimplantation embryos (sperm: 0.7 ± 0.5% and 1.8 ± 0.4%; oocytes: 15.0 ± 1.5% and 1.6 ± 0.9%; 4-cell embryos: 7.2 ± 1.1% and 0.5 ± 0.2%; and IVF blastocysts: 1.0 ± 0.5% and 0.9 ± 0.2%, respectively). These genes were also hypomethylated in bovine SCNT blastocysts (3.8 ± 1.7% and 0%) and BEFs (3.8 ± 1.5% and 1.8 ± 0.2%) (Figs 3 and 6). The BSP-PCR product of *CDX2* with BEFs genome, which has been treated with CpG MTase (M.SssI) enzyme, was used as positive control (95.3 ± 4.5%). *SOX2* and *CDX2* always showed significantly higher mRNA expression levels in oocytes, IVF preimplantation embryos and SCNT blastocysts than in BEFs (*P* < 0.01). The *SOX2* mRNA level was significantly lower in SCNT blastocysts than in IVF blastocysts (*P* < 0.01). However, there was no significant difference in *CDX2* mRNA levels between the SCNT and IVF blastocysts (*P* > 0.05) (Supplementary Fig. 6C,D).

### Influence of VC on locus-specific DNA methylation in bovine SCNT blastocysts

Previous studies have shown that VC can enhance somatic cell reprogramming during the generation of induced pluripotent stem cells (iPSCs)^25^. In this study, we explored the influence of VC on locus-specific DNA methylation in bovine SCNT blastocysts. We found that supplementation with VC (50 μg/ml) significantly increased the cleavage rate at 48 h (60.7 ± 3.5% and 77.7 ± 2.2%, *P* < 0.01) but did not influence the blastocyst rate on day 7 (21.0 ± 1.2% and 22.3 ± 0.9%, *P* > 0.05), the number of ICM cells per blastocyst (21.47 ± 3.95 and 20.23 ± 2.33, *P* > 0.05) and the total number of cells per blastocyst (92.73 ± 9.25 and 89.31 ± 10.42, *P* > 0.05) (Supplementary Table 2 and Supplementary Fig. 7).

**Figure 7:**
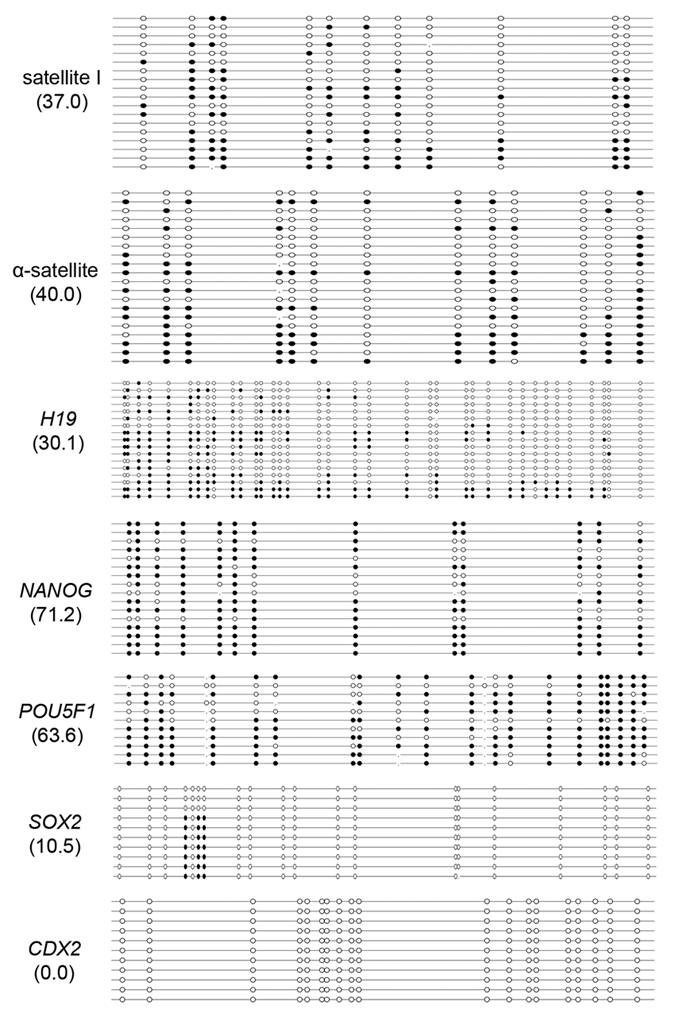
The influence of VC on locus-specific DNA methylation reprogramming. VC improved the DNA methylation status of the satellite I, α-satellite and *H19* genes and increased the methylation levels of *POU5F1* and *NANOG* in bovine SCNT blastocysts. Treatment with VC did not affect the methylation of *SOX2* or *CDX2*.

The results of bisulfite sequencing showed that treatment with VC influenced the reprogramming of locus-specific DNA methylation in bovine SCNT preimplantation embryos in a different way. Treatment with VC improved the methylation of satellite I (37.0 ± 1.7%), α-satellite (40.0 ± 2.5%) and *H19* (30.1 ± 3.1%) in bovine SCNT blastocysts, which are more closely related to bovine IVF blastocysts (Figs 3 and 7). Treatment with VC did not decrease the DNA methylation levels of the *NANOG* (71.2 ± 3.1%) and *POU5F1* (63.6 ± 3.5%) genes in bovine SCNT blastocysts (Figs 3 and 7), although it did significantly decrease mRNA expression levels in bovine SCNT blastocysts (*P* < 0.01) (Supplementary Fig. 8A,B). *SOX2* (10.5 ± 2.1%) and *CDX2* (0%) retained the same hypomethylation status in SCNT blastocysts treated with VC (Figs 3 and 7). Treatment with VC did not influence the mRNA expression level of *CDX2* (*P* > 0.05), but it did significantly increase the mRNA expression level of *SOX2* (*P* < 0.01) in SCNT blastocysts (Supplementary Fig. 8C,D).

## Discussion

A certain proportion of the embryos produced by nuclear transfer display developmental and metabolic abnormalities and have extremely low survival rates^30^. Studies of SCNT frequently cite the failure of the oocyte to properly reprogram the donor nucleus as the sole cause of the developmental defects^31^. The aberrant reprogramming of the methylation-controlled regions between the imprinted genes and satellite loci were found in cloned bovine fetuses^32^; however, the DNA methylation reprogramming patterns in bovine SCNT preimplantation embryos are not fully elucidated. In this study, the DNA methylation reprogramming patterns in bovine SCNT embryos were analyzed and compared with those in the IVF group.

IF staining for 5-mC and 5-hmC can reveal global DNA methylation reprogramming during the development of preimplanted mammalian embryos^13^,^33^. Previous research found that both 5-mC and 5-hmC existed in porcine IVF and SCNT preimplantation embryos^34^. Strong 5-mC signals were detected in both porcine IVF and SCNT embryos from the 2-cell stage to the 8-cell stage. There was a sudden decrease in the 5-mC signal within morula nuclei and a significant increase at the blastocyst stage. The dynamic changes in the 5-mC signals in bovine IVF and SCNT preimplantation embryos have also been reported by others; however, the other investigators did not simultaneously analyze the dynamic changes in the 5-hmC signals^23^,^35^. We simultaneously analyzed the dynamic changes in the 5-mC and 5-hmC signals, and our IF staining results showed that the 5-mC signals gradually decreased from the 2-cell to 8-cell embryos and then increased from the 8-cell embryos to the blastocysts in bovine IVF preimplantation embryos. The change in the 5-mC signal in bovine IVF preimplantation embryos reflects the typical pattern of demethylation and remethylation that is observed in mice^33^. The 5-hmC signals were observed in all of the developmental stages of the bovine IVF preimplantation embryos, indicating that DNA demethylation is actively occurring during the development of bovine IVF preimplantation embryos. The 5-mC signal was stronger in each developmental stage of the SCNT embryos than it was in the corresponding developmental stage of the IVF embryos, and no obvious change in the 5-mC signal was found in any developmental stage of the SCNT embryos. No 5-hmC signal was observed in any of the developmental stages of the bovine SCNT preimplantation embryos. Therefore, there was no or only slight DNA demethylation occurring during the development of bovine SCNT preimplantation embryos. The study on porcine IVF and SCNT embryos also found that the signal intensity of 5-mC in the SCNT preimplantation embryos was higher than that in their IVF counterparts; this phenomenon was also observed in our study on bovine IVF and SCNT embryos. The intensity of the 5-mC signal was higher in the inner cell mass (ICM) than in the TE regions, whereas the 5-hmC signal was uniformly distributed between the ICM and TE regions in porcine IVF blastocysts. In contrast, both the 5-mC and 5-hmC signals were symmetrically distributed between the ICM and TE cells in the porcine SCNT blastocyst. Our study found that 5-mC was symmetrically distributed between the ICM and TE cells in both bovine IVF and SCNT blastocysts and that 5-hmC was also uniformly distributed between the ICM and TE cells in bovine IVF blastocysts. However, no 5-hmC signals were observed in bovine SCNT blastocysts. Previous studies have found that the paternal genomic conversion of 5-mC into 5-hmC fails to occur and that the level of 5-mC remains constant in Tet3-deficient zygotes from conditional knockout mice^16^. Thus, the stronger 5-mC signals and the absence of 5-hmC might be caused by lower *TET3* expression levels in bovine SCNT preimplantation embryos (Supplementary Figs 2 and 4).

Aberrant locus methylation reprogramming was also found in bovine SCNT fetuses^32^. Repeat elements cover most parts of the mammalian genome, and functional genes only comprise approximately 1.5% of the entire genome^36^,^37^. Therefore, most of the 5-mC IF staining signals correspond to multiple-copy repetitive regions^38^. A study of mouse embryos found that some repeat sequences, such as intracisternal A particle (IAPs) elements, are exempted from complete DNA demethylation, while other repeat sequences, such as long interspersed elements (LINEs) and long terminal repeat (LTR) retroelements, are substantially demethylated during early embryonic development^39^. The aberrant reprogramming of the methylation of repeat elements in bovine SCNT preimplantation embryos has been found by *Acil* digestion^27^ and confirmed by our BSP-PCR results. Our results showed that the methylation levels in IVF blastocysts were lower than those in oocytes and sperm, indicating that a DNA demethylation process exists in satellite I and α-satellite during bovine IVF preimplantation embryonic development. However, satellite I and α-satellite were highly methylated in BEFs and SCNT blastocysts, and no obvious reduction was observed in SCNT blastocysts compared to BEFs. The fact that the methylation of multiple-copy elements reflects global genomic methylation may explain why the 5-mC signals in the SCNT embryos were stronger than they were in IVF embryos, and why no 5-hmC signals were found in SCNT embryos. The deregulation of imprinted genes caused abnormalities such as placental and fetal overgrowth and perinatal death in cloned animals^40^, and the differentially methylated regions (DMRs) at imprinted loci are resistant to the wave of active paternal and passive maternal DNA demethylation in the zygote and early preimplantation embryos of mice^41^ and pigs^29^. Our results found that the DMR in imprinted *H19* was highly methylated (near 100%) in sperm and almost completely unmethylated (near 0%) in oocytes. Similar results were detected within the DMR of the *IGF2* gene^42^. The DMR retained moderate DNA methylation in bovine IVF 4-cell embryos and blastocysts and high DNA methylation in BEFs. However, there was a slight decrease from gametes ((93.4 + 3.1)/2 = 48.25%) to 4-cell embryos (38.8 ± 1.9%) and an increase from 4-cell embryos to blastocysts (46.2 ± 4.5%). This dynamic change was also observed in the DMR region of the *IGF2* gene during bovine IVF preimplantation embryonic development^28^. In bovine SCNT blastocysts, the DMR was hypomethylated (10.5 ± 1.8%), and even lower methylation levels were detected in bovine IVF blastocysts (46.2 ± 4.5%), indicating that DNA demethylation occurs at the DMR during bovine SCNT preimplantation embryonic development. This DMR hypomethylation may cause the abnormal allelic expression of imprinted *H19*, which could induce abnormalities in cloned animals^43^. Given that *Dnmt1* was involved in the maintenance of methylation imprinting in cleavage-stage preimplantation embryos^44^,^45^, the low *DNMT1* mRNA levels in bovine SCNT preimplantation embryos may explain the DNA hypomethylation in the DMR of *H19* (Supplementary Fig. 3). Pluripotency genes, such as *POU5F1*, *NANOG*, *SOX2* and *CDX2*, are essential for the segregation and maintenance of embryonic and extraembryonic tissues, and the transcriptional regulation of these pluripotency genes is thought to be governed by epigenetic modifications such as DNA methylation^46^,^47^. *POU5F1* and *NANOG* followed a typical wave of DNA demethylation progression, whereas the CpG-rich regions of the *SOX2* and *CDX2* loci were hypomethylated throughout the development of porcine IVF preimplantation embryos^29^. The same DNA methylation reprogramming patterns of these pluripotency genes were found in our study of bovine IVF preimplantation embryos, and we also found a negative correlation between DNA methylation levels and the levels of *POU5F1* and *NANOG* transcription. *POU5F1* and *NANOG* were highly methylated in bovine SCNT blastocyst-stage embryos, and the abundance of *POU5F1* and *NANOG* mRNA was significantly lower than it was in IVF blastocyst-stage embryos (Supplementary Fig. 6). The low *TET3* mRNA levels in bovine SCNT preimplantation embryos may be the reason for the lack of demethylation of *POU5F1* and *NANOG* that inhibited the expression of these genes.

Vitamin C (VC), a general antioxidant, is responsible for maintaining the catalytic activity of a group of iron and 2-oxoglutarate-dependent dioxygenases^24^. Previous studies have shown that VC can enhance somatic cell reprogramming during the generation of induced pluripotent stem cells (iPSCs)^25^ and improve the *in vitro* and *in vivo* development of porcine SCNT embryos^26^. In contrast to previous reports^48^, our results showed that supplementation with VC (50 μg/ml) significantly increased the cleavage rate at 48 h (60.7 ± 3.5% and 77.7 ± 2.2%, *P* < 0.05) but did not influence the blastocyst formation rate on day 7 (21.0 ± 1.2% and 22.3 ± 0.9%, *P* > 0.05). We also found that VC had different effects on locus-specific DNA methylation reprogramming. VC improved the DNA demethylation of satellite I and α-satellite during bovine SCNT preimplantation embryonic development. It also increased the DNA methylation levels of *POU5F1* and *NANOG* and decreased the mRNA abundance in bovine SCNT blastocyst-stage embryos (Supplementary Fig. 8). Previous research found that VC has a vital role in determining the biological outcome of TET1 function at the cellular level^49^. Our results showed that *TET1* was mainly expressed in SCNT embryos after the 4-cell stage and that the demethylation of *POU5F1* and *NANOG* mainly occurred in IVF embryos after the 4-cell stage. These results may explain why VC did not improve the DNA demethylation of *POU5F1* and *NANOG* during bovine SCNT preimplantation embryonic development.

In summary, aberrant DNA methylation reprogramming was observed in bovine SCNT preimplantation embryos compared to IVF preimplantation embryos. Stronger 5-mC signals and no 5-hmC signals were found during bovine SCNT preimplantation embryonic development. The locus-specific (*POU5F1*, *NANOG*, satellite I, α-satellite and *H19*) reprogramming of DNA methylation was also abnormal in bovine SCNT preimplantation embryos. VC showed locus-specific effects on DNA methylation reprogramming in SCNT embryos. The aberrant DNA methylation reprogramming may be one of the reasons behind the frequent developmental and metabolic abnormalities and the extremely low survival rates of bovine SCNT embryos.

## Materials and Methods

### Chemicals and animals

All chemicals were purchased from Sigma-Aldrich (St. Louis, MO, USA), unless otherwise stated. All animal treatments were carried out in accordance with the experimental practices and standards approved by the Animal Welfare and Research Ethics Committee at Jilin University (Approval ID: 20151008-1).

### Sperm preparation and genomic DNA extraction

Bovine frozen-thawed semen was purchased from Ketian Co., Ltd, Changchun, China. The sperm were collected by centrifugation and incubated with D-PBS for 10 min at room temperature to remove somatic cell contamination. After centrifugation for 5 min at 10,000 g, the supernatant was discarded. The genomic DNA was extracted using the TIANamp Genomic DNA Kit (Tiangen, Beijing, China).

### Oocyte collection and *in vitro* maturation

Oocyte collection and *in vitro* maturation were similar to previous research^50^. Briefly, bovine ovaries were collected from a local abattoir and transported to the laboratory within 4 h, then cumulus-oocyte complexes (COCs) were aspirated from the follicles using an 18-gauge needle attached to a 10 ml syringe. The COCs with intact, unexpanded cumulus cells were cultured at 38.5 °C in 100 μl of maturation medium in a humidified 5% CO_2_ incubator. After 20 h of culture, the COCs were digested in 0.2% hyaluronidase for 3 min at 38.5 °C to separate all cumulus from oocytes. Oocytes with the first polar body (PB1) were considered matured and used for SCNT.

### Somatic cell nuclear transfer (SCNT)

Bovine embryo fibroblasts (BEFs) were used as donor cells for SCNT. BEFs were obtained from 2-day-old foetuses of Yanbian yellow cattle. For SCNT, cells were used within passages 3 to 7 at least 1 day after reaching confluence without serum starvation. The SCNT protocol was performed as previously described^51^. Briefly, oocytes with the first polar body were transferred into TCM199-Hepes medium containing 7.5 μg/ml cytochalasin B, and then, the first polar body and surrounding cytoplasm were removed using a bevelled pipette. Single cells were individually transferred to the perivitelline space of the recipient cytoplasts. Cell fusion was performed using two direct current pulses of 1.2 kV/cm for 10 μs by an Electro Cell Manipulator 2001 (BTX, San Diego, CA, USA) in 0.27 M mannitol, 0.1 mM CaCl_2_, 0.1 mM MgCl_2_, and 0.05% BSA. Fused eggs were activated with 5 μM ionomycin for 5 min at 24–25 h after the start of IVM, followed by treatment with 2 mM 6-DMAP in SOF medium containing 10% FBS for 4 h at 38.5 °C in 5% CO_2_ and 95% humidified air. After the activation, the eggs were then washed in SOF three times and cultured under mineral oil in 100 μl droplets of SOF with or without VC. The cleavage rates were determined 48 h after culturing, and the blastocyst rates were determined 7 days after culturing.

### Immunofluorescence (IF) staining

The zona pellucida (ZP) of bovine oocytes or embryos were removed by treatment with 0.5% pronase in TCM-199 at 38.5 °C for 5 min. Zona-free oocytes/embryos were briefly washed in PBS and fixed in 4% paraformaldehyde for 30 min at room temperature. After fixation, the oocytes/embryos were washed in PBS and permeabilized in PBS containing 0.1% Triton X-100 for 20 min. Then, the samples were divided into two groups. One group of samples was stained for TET3. To block the non-specific binding sites, the samples were incubated for 1 h at room temperature in PBS containing 0.01% Tween-20 and 2% bovine serum albumin (BSA); this was followed by an incubation in blocking solution together with primary antibody raised against TET3 (dilution 1:50; Santa) overnight at 4 °C. The next day, the oocytes/embryos were washed in blocking solution and stained with Alexa Fluor 488 goat anti-rabbit (1:200; Invitrogen), which could recognize primary antibodies for TET3, for 1 h at room temperature. DNA was stained with 10 μg/ml Hoechst 33342 for 10 min. Another group of samples was stained for 5 mC/5 hmC. After permeabilization, the samples were then treated with 4N HCl for 30 min at room temperature and subsequently neutralized for 10 min with 100 mM Tris-HCl buffer, pH 8.5. Then, the samples were incubated for 1 h in blocking solution, followed by incubation with primary antibodies, anti-5 mC (1:100; Eurogentec) or anti-5 hmC (1:100; Active Motif) overnight at 4 °C. The next day, the samples were incubated with secondary antibodies, Alexa Fluor 568 goat anti-mouse (1:200; Invitrogen) and Alexa Fluor 488 goat anti-rabbit (1:200; Invitrogen), for 1 h at room temperature. All samples were mounted between a cover slip and a glass slide supported by four columns of a mixture of petroleum jelly and paraffin (9:1) and immediately observed under a fluorescent microscope (Nikon, Tokyo, Japan) equipped with a digital camera. All images were captured with the same exposure times and microscope settings. At least 5 embryos at each development stage were analyzed. The fluorescence intensity was quantified by the Imageproplus 6.0.

### RNA isolation, cDNA preparation and qRT-PCR

At least thirty bovine oocytes or blastomeres of IVF and SCNT embryos was used to extract all RNA using the RNeasy Mini kit (Qiagen, Hilden, Germany). The 1st-Strand cDNA Synthesis kit (Promega, Madison, WI, USA) was used to synthesize the first-strand cDNA. The primers used for qRT-PCR analysis are listed in Supplementary Table 1. The real-time PCR mix (20 μl) consisted of 2 μl of cDNA, 10 μl of SYBR green master mix, 6.4 μl of RNase-free water and 0.8 μl each of forward and reverse primers (10 pmol) for each gene. The programme used for the amplification of all genes consisted of a denaturing cycle of 3 min at 95 °C, 40 cycles of PCR (95 °C for 10 s, 55 °C for 45 s, and 95 °C for 1 min), a melting curve analysis consisting of 95 °C for 1 min followed by 55 °C for 1 min, a step cycle starting at 55 °C for 10 s with a 0.5 °C/s transition rate, and cooling at 4 °C. Relative gene expression data were analysed using Quantitative Real-Time PCR (qRT-PCR) and the 2^−ΔΔCT^ method. The qRT-PCR analysis were performed three times for each sample.

### Sodium bisulfite genomic sequencing

Bisulfite sequencing was used to analyse locus-specific DNA methylation of BEFs, oocyte, IVF and SCNT blastocysts as described^52^. Briefly, at least fifty cells or blastomeres were treated with a lysis solution (10 mM Tris-HCl, pH 7.6, 10 mM EDTA, 1% SDS, and 20 μg/μl of proteinase K in ddH_2_O) for 1.5 h at 37 °C. Then, the mixture was boiled for 5 min in a water bath, chilled on ice and quickly spun down. Then, 4 μl of 2 M N_a_OH (final concentration 0.3 M N_a_OH) was added, and the mixture was incubated at 15 min at 50 °C. Samples were mixed with 2 volumes of 2% low melting point agarose and pipetted into chilled mineral oil to form beads. Then, the beads were treated with freshly made bisulfite solution (2.5 M sodium metabisulfite and 125 mM hydroquinone, pH 5) for 5 h in the dark and covered with mineral oil at 50 °C. The reactions were stopped by equilibration against 1 ml of Tris-EDTA buffer (pH 8.0) for 4 × 15 min. After desulfonation in 0.5 ml 0.2 M N_a_OH for 2 × 15 min, the beads were washed with 1 ml Tris-EDTA buffer for 3 × 10 min and H_2_O for 2 × 15 min, and then used for PCR. The PCR primer sequences were listed in Supplementary Table 1. The purified PCR fragments were then cloned into a Pmd^TM^18-T vector for sequencing (TaKaRa, Japan). The PCR amplifications and subsequent sequencing were performed three times for each sample. At least 10 clones per gene were sequenced. DNA methylation situations from 0% to 20% was considered as low DNA methylation level, from 21% to 50% was considered as moderate DNA methylation level, from 51% to 1000% was considered as high DNA methylation level in our bisulfite sequence analysis.

### Statistical analysis

At least three technical replicates and biological replicates for each data analysis. Data were analyzed with Statistics Production for Service Solution (Version 16.0; SPSS, Chicago, IL, USA) by one-way ANOVA. A value of *P* < 0.05 was considered different, and *P* < 0.01 was considered significantly different.

## Additional Information

**How to cite this article**: Zhang, S. *et al*. Aberrant DNA methylation reprogramming in bovine SCNT preimplantation embryos. *Sci. Rep*. **6**, 30345; doi: 10.1038/srep30345 (2016).

## Supplementary Material

Supplementary Information
