# Supplementary material for: Aberrant DNA methylation reprogramming in bovine SCNT preimplantation embryos
Source: Sci Rep. 2016 Jul 26;6:30345. doi: 10.1038/srep30345 (PMC4960566; doi:10.1038/srep30345)
Supplement: Supplementary Information [file srep30345-s1.doc]

**Aberrant DNA methylation reprogramming in bovine SCNT preimplantation embryos**

**Sheng Zhang^1,3†^, Xin Chen^2†^, Fang Wang^3^, Xinglan An^3^, Bo Tang^3^, Xueming Zhang^3^, Liguang Sun^1^, Ziyi Li^1^***

^1^ State & Local Joint Engineering Laboratory for Animal Models of Human Diseases, Academy of Translational Medicine, First Hospital, Jilin University, Changchun, Jilin130061, China

^2^ Air Force General Hospital of PLA, Beijing 100037, China

^3^ College of Animal Science and Veterinary Medicine, Jilin University, Changchun, Jilin130062, China

**^†^These authors contributed equally to this work**

**Correspondence author:**

Ziyi Li, Ph.D., E-mail: ziyi@jlu.edu.cn

**Relative expression of *MMP3* in BEFs and SCNT blastocysts**

To confirm the lost expression of fibroblast-specific markers in SCNT embryos, we analyzed the expression of *MMP3* in BEFs and SCNT blastocysts. The results showed that there was almost no *MMP3* expression in SCNT blastocysts when compared with BEFs (Supplementary Fig. 1).


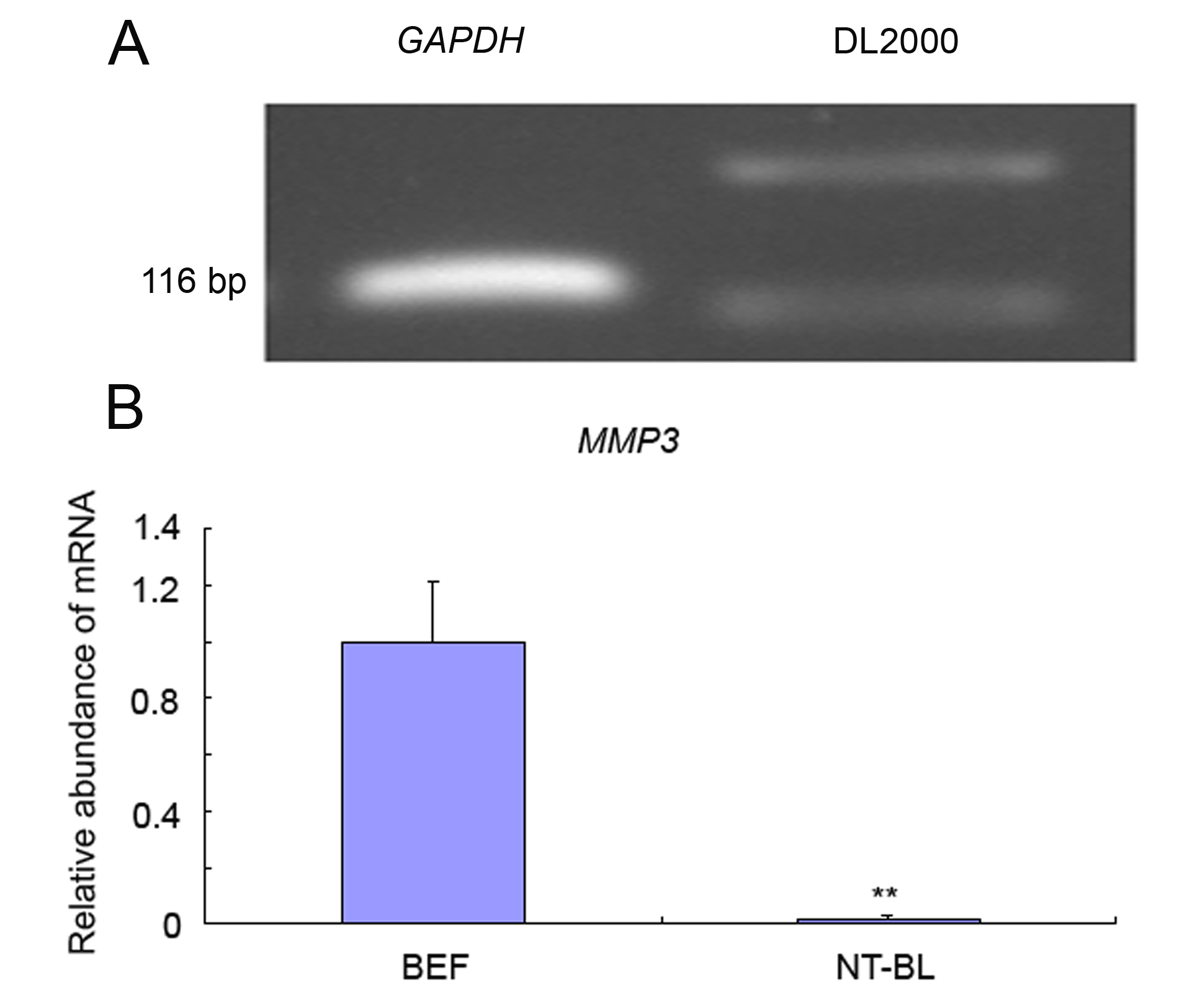


**Figure S1. The relative abundance of *MMP3* mRNA in BEFs and SCNT blastocysts.** *GAPDH* was used as a normalizer. The expression level of *MMP3* in BEFs was used as a calibrator (expression set to 1). The results represent the mean ± standard deviation of three independent experiments in which at least three SCNT blastocysts were used to extract total RNA. ** *P* < 0.01.

**Relative expression of *TET* transcripts in bovine IVF and SCNT preimplantation embryos**

A high expression of *TET3* was observed in oocytes and IVF embryos at the 2-cell and 4-cell stages, and a significantly lower level of *TET3* expression was observed in IVF blastocysts. However, *TET3* showed significantly lower expression in SCNT embryos at the 2-cell stage when compared with IVF embryos (*P* < 0.01). The expression level was further reduced following the development of SCNT preimplantation embryos, while no *TET3* expression was observed in the blastocysts of SCNT embryos. No expression of *TET1* was observed in oocytes or in the 2- or 4-cell-stage IVF embryos. The expression of *TET1* was observed in blastocyst-stage IVF embryos, albeit at very low levels. In SCNT preimplantation embryos, the expression of *TET1* was observed at the 2-cell stage, and the level increased until the blastocyst stage. *TET2* expression remained low in both IVF and SCNT preimplantation embryos (Supplementary Fig. 2).
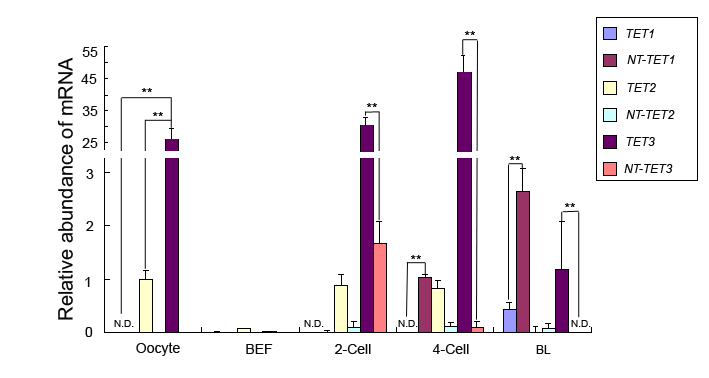


**Figure S2.** **The relative abundance of *TET1*, *TET2* and *TET3* mRNA in bovine oocytes, IVF and SCNT preimplantation embryos and BEFs.** The levels of the bovine *TET* family members are shown. 18S rRNA was used as a normalizer. The level of *TET2* expression in oocytes was used as a calibrator (expression set to 1). The expression levels of the *TET* family members at each IVF embryonic stage were compared to the levels in the corresponding stages of SCNT embryos. N.D.: no data; BL: blastocysts; BEFs: bovine embryonic fibroblasts; *NT-TET1*: *TET1* gene in SCNT preimplantation embryos; *NT-TET2*: *TET2* gene in SCNT preimplantation embryos; and *NT-TET3*: *TET3* gene in SCNT preimplantation embryos. The results represent the mean ± standard deviation of three independent experiments in which at least thirty bovine oocytes or blastomeres of IVF or SCNT embryos were used to extract total RNA. ** *P* < 0.01.

**Relative expression of *DNMT* transcripts in bovine IVF and SCNT preimplantation embryos**

The patterns of dynamic change in the expression of *DNMT* gene family members were similar between IVF and SCNT preimplantation embryos, but they were lower in SCNT embryos than in IVF preimplantation embryos (Supplementary Fig. 3). In oocytes and during the early developmental stages of IVF and SCNT preimplantation embryos, a high expression of *DNMT1* and *DNMT3b* were observed. At the blastocyst stage, the expression level of *DNMT1* decreased and was significantly lower than that in oocytes (*P* < 0.01). However, *DNMT3b* still maintained higher expression in IVF and SCNT blastocysts. *DNMT3a* maintained lower expression levels in oocytes and during all of the developmental stages of IVF and SCNT preimplantation embryos.


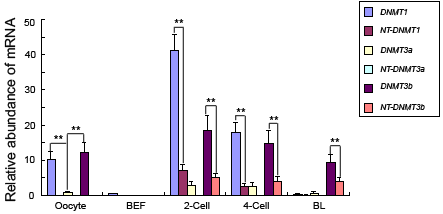


**Figure S3.** **The relative abundance of *DNMT1*, *DNMT3a* and *DNMT3b* mRNA in bovine oocytes, IVF and SCNT preimplantation embryos and BEFs.** The levels of the bovine *DNMT* family members are shown. 18S rRNA was used as a normalizer. The *DNMT3a* expression level in oocytes was used as a calibrator (expression set to 1). The expression levels of the *DNMT* family members at each IVF embryonic stage were compared to the levels of the *DNMT* family members in the corresponding stages of SCNT embryos. BL: blastocysts; BEFs: bovine embryonic fibroblasts; *NT-DNMT1*: *DNMT1* gene in SCNT preimplantation embryos; *NT-DNMT3a*: *DNMT3a* gene in SCNT preimplantation embryos; and *NT-DNMT3b*: *DNMT3b* gene in SCNT preimplantation embryos. The results represent the mean ± standard deviation of three independent experiments in which at least thirty bovine oocytes or blastomeres of IVF or SCNT embryos were used to extract total RNA. ** *P* < 0.01.

**Staining of TET3 in IVF and SCNT preimplantation embryos**

The expression and subcellular localization of TET3 in oocytes, bovine IVF and SCNT preimplantation embryos were analyzed using TET3 immunofluorescent (IF) staining. We observed TET3 signals in the cytoplasm of oocytes. These signals became stronger and localized to the nucleus of IVF embryos from the 2-cell stage to the 8-cell stage (Supplementary Fig. 4A and 4C). After the 8-cell stage, they gradually became weaker until they disappeared in IVF embryos at the blastocyst stage. Compared to IVF embryos, SCNT preimplantation embryos exhibited weaker signals in each stage of bovine development; the signals also appeared in the cytoplasm (Supplementary Fig. 4B and 4C).


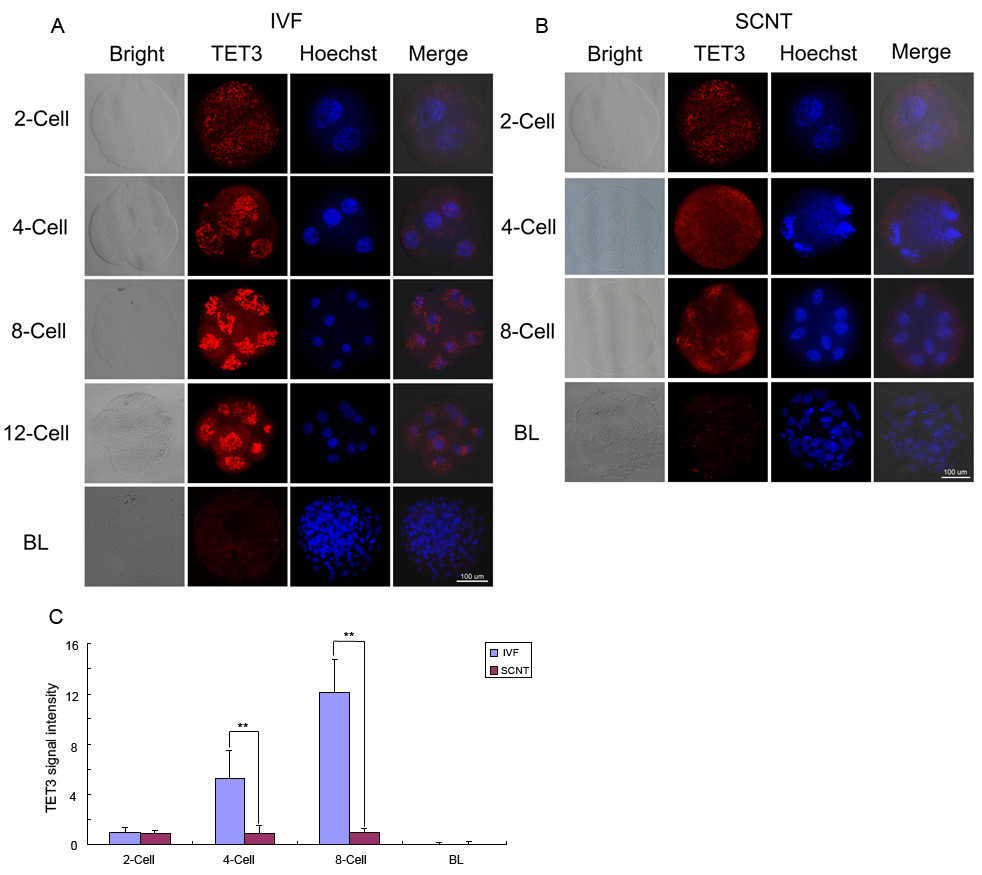


**Figure S4.** **Immunofluorescent (IF) staining of TET3 in bovine oocytes and IVF and SCNT preimplantation embryos.** (A) The distribution of TET3 (red) in bovine oocytes and IVF preimplantation embryos. (B) The distribution of TET3 in bovine SCNT preimplantation embryos. (C) The TET3 signal intensities were measured in each embryo. The signal intensity in the IVF 2-cell embryo was used as a calibrator (set to 1). At least 5 embryos at each developmental stage were analyzed. The DNA was stained with Hoechst. The results represent the mean ± standard deviation of five independent experiments. ** *P* < 0.01. BL: IVF blastocysts.

**Methylation of satellite I in granulosa cells and SCNT blastocysts**

We used granulosa cells as the donor cells for SCNT and collected the blastocyst (G-NT-BL). The methylation of satellite I in granulosa cells and G-NT-BL was analyzed. The results showed that satellite I was highly methylated in bovine granulosa cells (88.0 ± 5.6%) and G-NT-BL (82.3% ± 3.7%) (Supplementary Fig. 5).


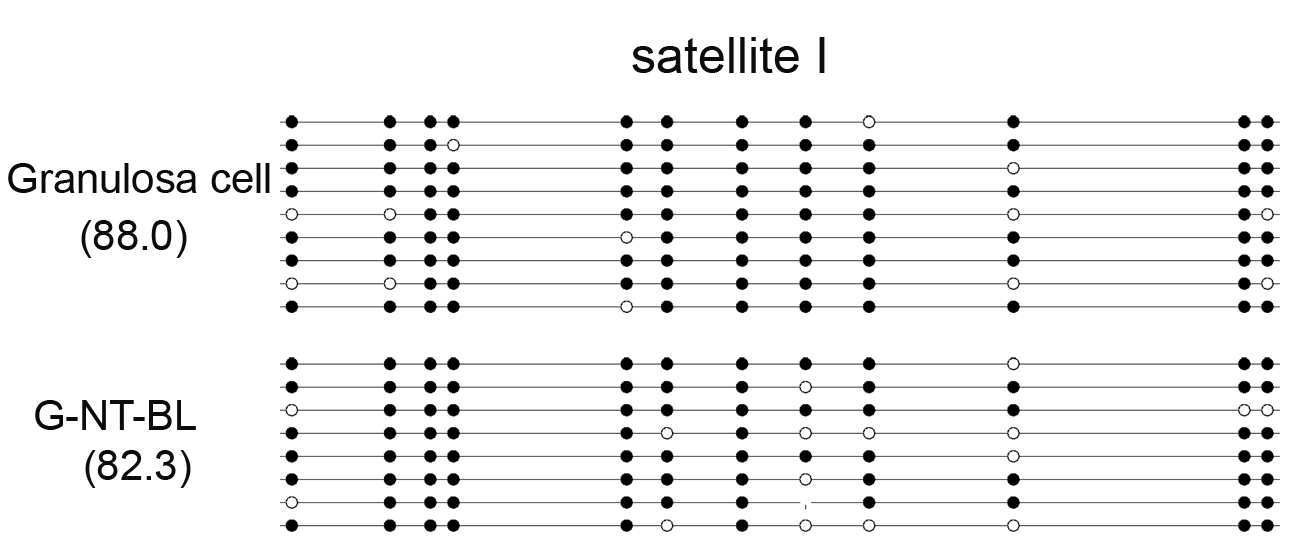


**Figure S5.** **DNA methylation profile of satellite I in bovine granulosa cells and G-NT-BL.** Satellite I was hypermethylated in both granulosa cells and G-NT-BL. G-NT-BL: SCNT blastocysts from granulosa donor cells.

**Relative expression of pluripotency genes in oocytes, IVF and SCNT preimplantation embryos and BEFs**

*POU5F1* and *NANOG* were highly expressed in IVF and SCNT embryos at the blastocyst stage; however, the expression levels in SCNT embryos at the blastocyst stage were significantly lower than those in IVF embryos at the blastocyst stage (Supplementary Fig. 6A and 6B). *SOX2* and *CDX2* mRNA expression levels were always high in oocytes, bovine IVF preimplantation embryos and SCNT embryos at the blastocyst stage. *SOX2* mRNA levels were significantly lower in SCNT embryos compared to IVF embryos at the blastocyst stage (*P* < 0.01). There was no difference in *CDX2* mRNA levels in the SCNT and IVF embryos at the blastocyst stage (*P* > 0.05) (Supplementary Fig. 6C and 6D). All of the pluripotency genes that were studied were expressed at lower levels in BEFs.


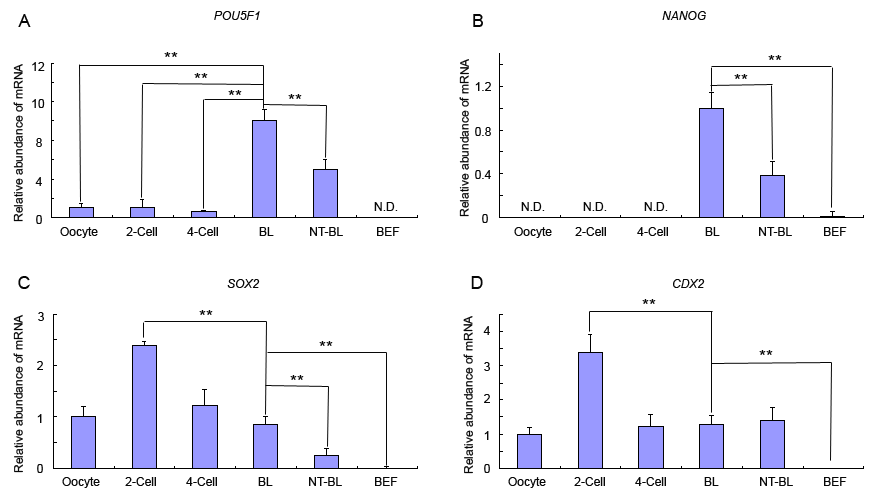


**Figure S6. The relative abundance of *POU5F1*, *NANOG*, *SOX2* and *CDX2* mRNA in bovine oocytes, IVF and SCNT preimplantation embryos and BEFs.** 18S rRNA was used as a normalizer. The expression levels of *POU5F1*, *SOX2* and *CDX2* in oocytes and the expression level of *NANOG* in IVF blastocyst-stage embryos were used as calibrators (expression set to 1), and the expression level of the gene at each stage was compared to the level of the gene in IVF blastocyst-stage embryos. (A) The relative abundance of *POU5F1* mRNA in bovine oocytes, IVF and SCNT preimplantation embryos and BEFs. (B) The relative abundance of *NANOG* mRNA in bovine oocytes, IVF and SCNT preimplantation embryos and BEFs. (C) The relative abundance of *SOX2* mRNA in bovine oocytes, IVF and SCNT preimplantation embryos and BEFs. (D) The relative abundance of *CDX2* mRNA in bovine oocytes, IVF and SCNT preimplantation embryos and BEFs. N.D.: no data; BL: IVF blastocysts; NT-BL: SCNT blastocysts; and BEFs: bovine embryonic fibroblasts. The results represent the mean ± standard deviation of three independent experiments in which at least thirty bovine oocytes or blastomeres of IVF or SCNT embryos were used to extract total RNA. ** *P* < 0.01.

**The influence of VC on the developmental capacity of bovine SCNT preimplantation embryos**

We found that supplementation with VC (20 µg/ml) did not signiﬁcantly influence the cleavage rate at 48 h (60.7 ± 3.5% and 60.3 ± 3.8%, *P* > 0.05), the blastocyst rate on day 7 (21.0 ± 1.2% and 23.0 ± 1.0%, *P* > 0.05), the number of ICM cells per blastocyst (21.47 ± 3.95 and 21.74 ± 2.17, *P* > 0.05) or the total number of cells per blastocyst (92.73 ± 9.25 and 91.86 ± 11.74, *P* > 0.05). Supplementation with VC (50 µg/ml) signiﬁcantly increased the cleavage rate at 48 h (60.7 ± 3.5% and 77.7 ± 2.2%, *P* < 0.01) but did not influence the blastocyst rate on day 7 (21.0 ± 1.2% and 22.3 ± 0.9%, *P* > 0.05), the number of ICM cells per blastocyst (21.47 ± 3.95 and 20.23 ± 2.33, *P* > 0.05) or the total number of cells per blastocyst (92.73 ± 9.25 and 89.31 ± 10.42, *P* > 0.05). Supplementation with VC (100 µg/ml) signiﬁcantly increased the cleavage rate at 48 h (60.7 ± 3.5% and 76.3 ± 0.8%, *P* < 0.01) and signiﬁcantly decreased the blastocyst rate on day 7 (21.0 ± 1.2% and 14.3 ± 0.9%, *P* < 0.01), the number of ICM cells per blastocyst (21.47 ± 3.95 and 14.51 ± 3.89, *P* < 0.01) and the total number of cells per blastocyst (92.73 ± 9.25 and 72.47 ± 8.61, *P* < 0.01) (Supplementary Table 2) (Supplementary Fig. 7).


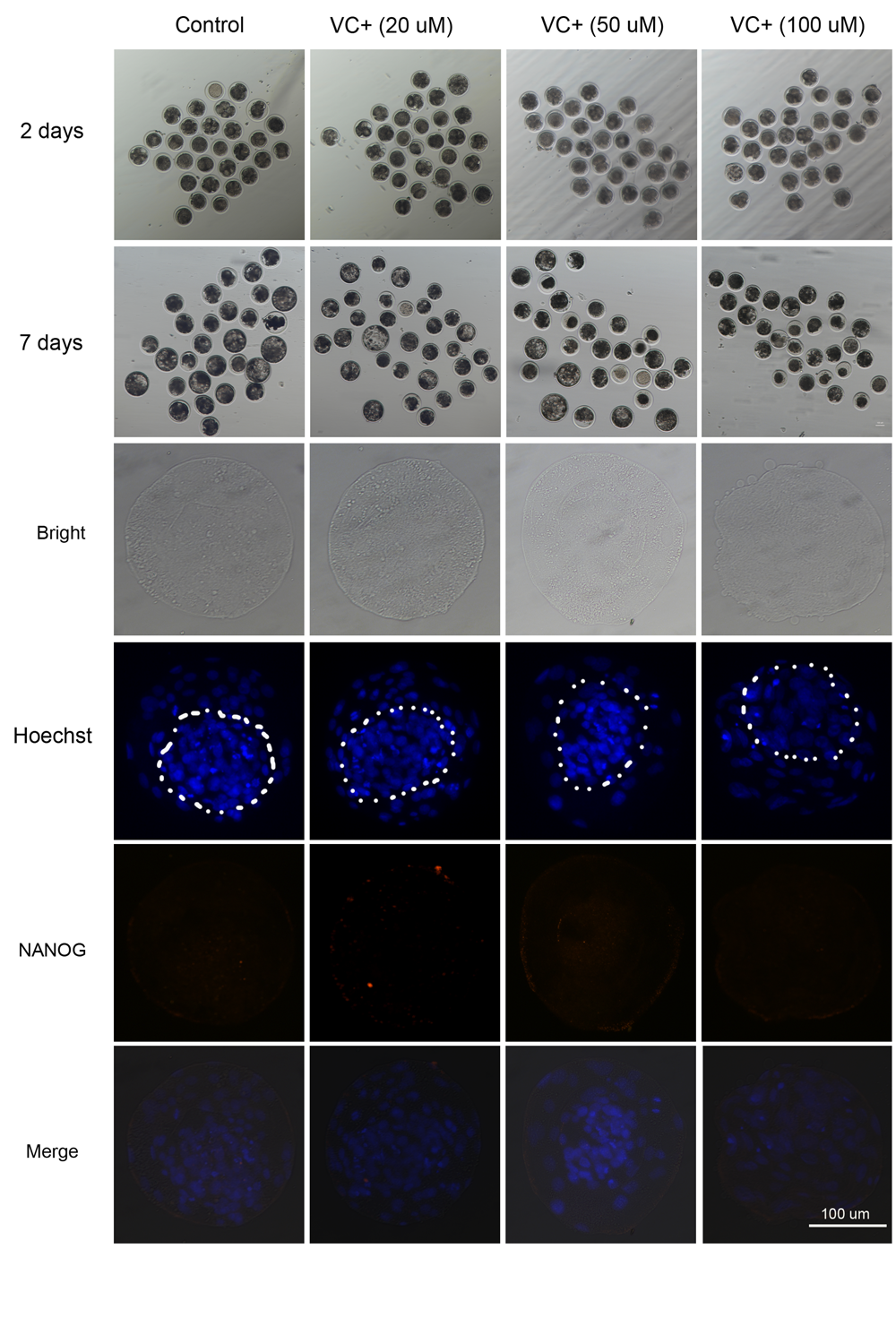


**Figure S7. The influence of VC on the developmental capacity of bovine SCNT preimplantation embryos.** The cleavage rates were determined 2 days after culturing, and the blastocyst rates were determined 7 days after culturing. At least five blastocysts per group were stained with NANOG (red) and Hoechst, and the number of ICM cells and total cells were then counted. The white, dashed circle denotes the inner cell mass.

**The influence of VC on the expression of pluripotency genes in SCNT blastocysts**

Treatment with VC had a different effect on the expression of pluripotency genes. VC significantly decreased the mRNA expression levels of *POU5F1* and *NANOG* in SCNT embryos at the blastocyst stage (*P* < 0.01) and significantly increased the mRNA expression level of *SOX2* (*P* < 0.01). However, it did not influence the mRNA expression level of *CDX2* in SCNT embryos at the blastocyst stage (Supplementary Fig. 8) (*P* > 0.05).


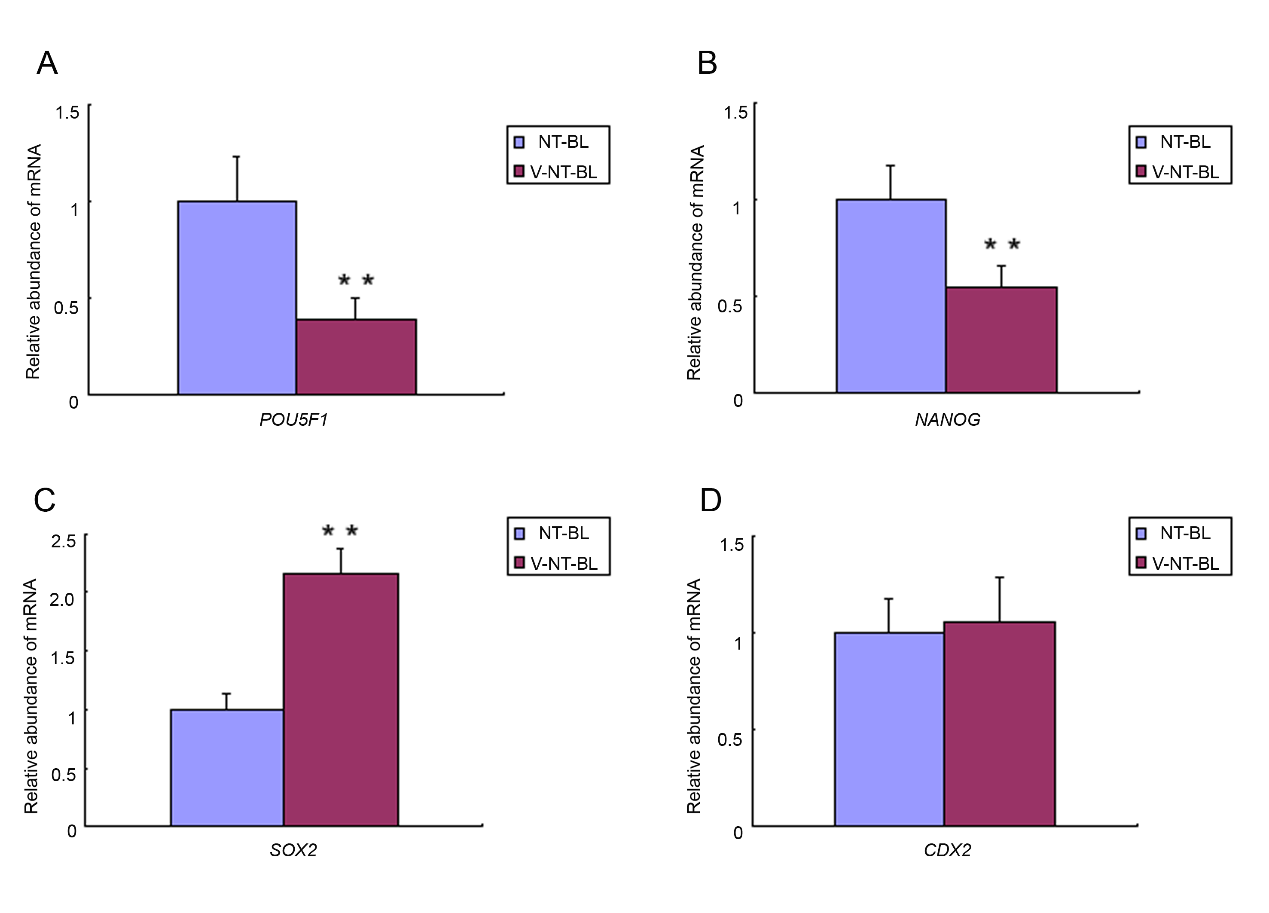
 **Figure S8. The relative abundance of *POU5F1*, *NANOG*, *SOX2* and *CDX2* mRNA in untreated and VC-treated bovine SCNT blastocyst-stage embryos.** 18S rRNA was used as a normalizer. The expression level of the gene in SCNT blastocyst-stage embryos was used as a calibrator (expression set to 1), and the expression level of the gene in blastocyst-stage embryos treated with VC was compared to the level of the gene in untreated SCNT blastocyst-stage embryos. (A) The relative abundance of *POU5F1* mRNA. (B) The relative abundance of *NANOG* mRNA. (C) The relative abundance of *SOX2* mRNA. (D) The relative abundance of *CDX2* mRNA. NT-BL: SCNT blastocysts; and V-NT-BL: SCNT blastocysts treated with VC. The results represent the mean ± standard deviation of three independent experiments in which at least thirty bovine oocytes or blastomeres of IVF or SCNT embryos were used to extract total RNA. ** *P* < 0.01

**Table S1.** Primers for qRT-PCR and Bisulfite-sequencing PCR

| Primer | Primer pair sequences ( 5’ to 3’ )^*^ | Product size （ bp ） |
| --- | --- | --- |
| qRT-PCR  18S rRNA  *GAPDH*  *TET1*  *TET2*  *TET3*  *DNMT1*  *DNMT3a*  *DNMT3b*  *NANOG*  *POU5F1*  *SOX2*  *CDX2*  *MMP3*  Bisulfite-sequencing PCR  *NANOG*  *POU5F1*  *SOX2*  *CDX2*  *H19*  α-satellite  satellite I | F:GACTCATTGGCCCTGTAATTGGAATGAGTC  R: GCTGCTGGCACCAGACTTG  F:GCAAGTTCAACGGCACAGTCA  R:AGCACCAGCATCACCCCAC  F: TTCCCACGGCTCGGTTCT  R: RTTTCTGTTCGGAGGCTTTAGTTT  F:AAGGCTGAGGGACGAGAACGA  R:GAGACGGAGATGGTATCAAGAATGG  F: CCCTCCTGAAGAGCACCCATCC  R: TCGGGCCGCTTGAATACTGACT  F: TGACTCCACCTACGAAGACC  R: TCTCTACTTGCTCCACCACG  F: CAACGGAGAAGCCTAAGGTCAA  R: TTGA GGCTCCCACAAGAGATG  F: AGTATCAGGATGGGAAGGAGTTTG  R: CCAGGAGA AACCCTTGATCTTTC  F: AACAACTGGCCGAGGAATAG  R: AGGAGTGGTTGCTCCAAGAC  F:GGCGCCAGAGGAAAGGATAC  R:AGAAGGGCAAACGATCAAGCA  F: CTATGACCAGCT CGCAGA  R: GGAAGAAGAGGTAACCACG  F: AGACAAATACCGGGTCGTGTACA  R: TTTGCTCTGCGGTTCTGAA  F:CCTTGTTGCTGCCCCATGAACTT  R:TGAGAAAGGCGGAACCGAGTG  F: TTTTTTAATTATAATTTGATGGGGT  R: CTAACACACCTTAAATAAACAAACC  F: GATTTGGATGAGTTTTTAAGGGTT  R: ACTCCAACTTCTCCTTATCCAACTT  F: AGAAGGTTTTGAGGATAGAATTTT  R: ACAACCTACCTACCAACCACTA  F: TGGAAGGAGGAAGTTTTTAATA  R: CACCTCCTTTCCACTAAACTAC  F: TTAAGGTTTTGGTTTTTGTTT  R: AACTTCAAAATTACCTCCTACC  F: AATAATTCCACATTCCRTAAAACCC  R: GATGTTTYGGGGAGAGAGG  F: AATACCTCTAATTTCAAACT  R:TTTGTGAATGTAGTTAATA | 87  116  214  285  219  128  121  74  193  173  152  162  120  288  293  441  291  390  189  211 |

* F, forward; R, reverse.

**Table S2.** Bovine SCNT Embryo cleavage rates and blastocyst rates following culture supplement with VC

| Group | No. of embryos  examined | | No. of embryos  cleaved ( % ) | No. of embryos developed to the blastocyst stage ( % ) | Total No. of ICM cells per blastocyst | Total No. of cells per blastocyst |
| --- | --- | --- | --- | --- | --- | --- |
| Control (0 µM)  VC (20 µM)  VC (50 µM)  VC (100 µM) | 150  153  161  120 | 91 (60.7 ± 3.5)^a^  92 (60.3 ± 3.8)^a^  125 (77.7 ± 2.2)^b^  167 (76.3 ± 0.8)^b^ | | 20 (21.0 ± 1.2)^a^  21 (23.0 ± 1.0)^a^  28 (22.3 ± 0.9)^a^  17 (14.3 ± 0.9)^b^ | 21.47 ± 3.95^a^  21.74 ± 2.17^a^  20.23 ± 2.33^a^  14.51 ± 3.89^b^ | 92.73 ± 9.25^a^  91.86 ± 11.74^a^  89.31 ± 10.42^a^  72.47 ± 8.61^b^ |

Data are the mean ± SEM of at least three trials. Values in the same column with different superscripts differ significantly (*P* < 0.05).
